# Reconstitution of Pure Chaperonin Hetero-Oligomer Preparations *in Vitro* by Temperature Modulation

**DOI:** 10.3389/fmolb.2018.00005

**Published:** 2018-01-26

**Authors:** Anna Vitlin Gruber, Milena Vugman, Abdussalam Azem, Celeste E. Weiss

**Affiliations:** ^1^Department of Molecular, Cell and Developmental Biology, University of California, Los Angeles, Los Angeles, CA, United States; ^2^Department of Biochemistry and Molecular Biology, George S. Wise Faculty of Life Sciences, Tel Aviv University, Tel Aviv, Israel

**Keywords:** chaperone, chaperonin, chloroplast, *A. thaliana*, oligomer, *in vitro*, temperature

## Abstract

Chaperonins are large, essential, oligomers that facilitate protein folding in chloroplasts, mitochondria, and eubacteria. Plant chloroplast chaperonins are comprised of multiple homologous subunits that exhibit unique properties. We previously characterized homogeneous, reconstituted, chloroplast-chaperonin oligomers *in vitro*, each composed of one of three highly homologous beta subunits from *A. thaliana*. In the current work, we describe alpha-type subunits from the same species and investigate their interaction with β subtypes. Neither alpha subunit was capable of forming higher-order oligomers on its own. When combined with β subunits in the presence of Mg-ATP, only the α2 subunit was able to form stable functional hetero-oligomers, which were capable of refolding denatured protein with native chloroplast co-chaperonins. Since β oligomers were able to oligomerize in the absence of α, we sought conditions under which αβ hetero-oligomers could be produced without contamination of β homo-oligomers. We found that β2 subunits are unable to oligomerize at low temperatures and used this property to obtain homogenous preparations of functional α2β2 hetero-oligomers. The results of this study highlight the importance of reaction conditions such as temperature and concentration for the reconstitution of chloroplast chaperonin oligomers *in vitro*.

## Introduction

Chaperonins are a subfamily of chaperone proteins found in bacteria and bacteria-derived organelles. In contrast to the well-studied GroEL of *Escherichia coli*, which has one Cpn60 gene product (Johnson et al., 1989), that forms functional homo-oligomers composed of 14 subunits, chloroplasts contain two Cpn60 subtypes, Cpn60α and Cpn60β (Musgrove et al., 1987; Martel et al., 1990; Cloney et al., 1992a,b, 1993, 1994; Nishio et al., 1999). These subtypes exhibit ~50% homology to each other, similar to their respective homologies to GroEL, and are each present in two or more paralogous forms in most higher plants (Hemmingsen et al., 1988; Cloney et al., 1994; Hill and Hemmingsen, 2001). These subunits combine to form extremely labile hetero-oligomeric chaperonin species, which dissociate into monomeric form upon dilution, particularly in the presence of ATP (Musgrove et al., 1987; Roy et al., 1988; Lissin, 1995; Viitanen et al., 1998; Dickson et al., 2000; Bonshtien et al., 2009).

*Arabidopsis* chloroplast contains six Cpn60 homologs: two Cpn60α subunits and four Cpn60β subunits (Hill and Hemmingsen, 2001). Unlike Cpn60β proteins which share a high level of sequence similarity (Vitlin et al., 2011), significant divergence of primary structure is apparent between the two Cpn60α paralogs. The two *Arabidopsis* Cpn60α proteins are similar in length (543 and 541 amino acids) and share 60% identity of peptide sequence (excluding the putative transit peptide). The sequence differences are evenly distributed along the length of the proteins (Hill and Hemmingsen, 2001).

Many species contain orthologs of both Cpn60α1 (At5g18820) and Cpn60α2 (At2g28000). Several groups characterized knock-out or point mutants of Cpn60α2 orthologs, all resulting in severe impairment of plant development (Apuya et al., 2001; Suzuki et al., 2009; Peng et al., 2011; Feiz et al., 2012; Kim et al., 2013; Jiang et al., 2014; Ke et al., 2017). A knockout strain of α1 was arrested at the globular embryo stage (Ke et al., 2017), while an α2 knockout was arrested at the heart stage (Apuya et al., 2001). Cpn60α1 and Cpn60α2 vary greatly in their expression levels. Cpn60α2 was shown to be the most highly expressed of the Cpn60 homologs in all tissues and during all developmental stages in comparison with other chaperonins (Weiss et al., 2009). In contrast, Cpn60α1 subunit expression is barely detectable at the RNA level (Weiss et al., 2009) although recent studies reported that this protein is highly expressed in the SAM of early seedlings and embryonic cotyledons (Ke et al., 2017).

Several groups have investigated the oligomerization of chloroplast Cpn60 subunits from different plants *in vitro*. Attempts to reconstitute oligomers from purified *P. sativum* α monomers alone were unsuccessful. However, upon addition of β subunits, hetero-oligomers were formed, composed of α and β subunits (αβ hetero-oligomer) (Dickson et al., 2000). These results were consistent with studies on chaperonins from *Brassica napus* and *C. reinhardtii*, which produced functional αβ oligomers when over-expressed together in *E. coli* (Cloney et al., 1992a,b; Bai et al., 2015). Similar to GroEL, reconstituted αβ hetero-oligomers from *P. sativum* could mediate the refolding of denatured substrate when assisted with co-chaperonin from any source: bacteria (GroES), mitochondria (mt-cpn10) or chloroplast (Cpn20) (Dickson et al., 2000), whereas beta homo-oligomers were functional *in vitro* with native chloroplast co-chaperonins and with heterologous mt-cpn10 (Dickson et al., 2000; Vitlin et al., 2011).

In this work we used a well-established method for Cpn60 monomer purification and oligomer reconstitution, that was developed in our lab (Vitlin et al., 2011), in order to study both *Arabidopsis* Cpn60α subunits as monomers, as well as the hetero-oligomers that are formed together with Cpn60β subunits. We show that the α2 subunit can form functional oligomers with β subunits, while the α1 subunit is unable to oligomerize under any conditions that we tested *in vitro*. Since β subunits oligomerize on their own, production of pure αβ hetero-oligomer is liable to be contaminated by β homo-oligomer. The dependence of reconstitution on temperature and concentration can be manipulated to ensure that the resulting hetero-oligomeric preparations are homogeneous. In this work, we present a method for reconstitution of hetero-oligomers, composed of α2 and β2 subunits that are free of contaminating β2 homo-oligomers.

## Results

### Purification and structural characterization of alpha subunits

**Figure 1:**
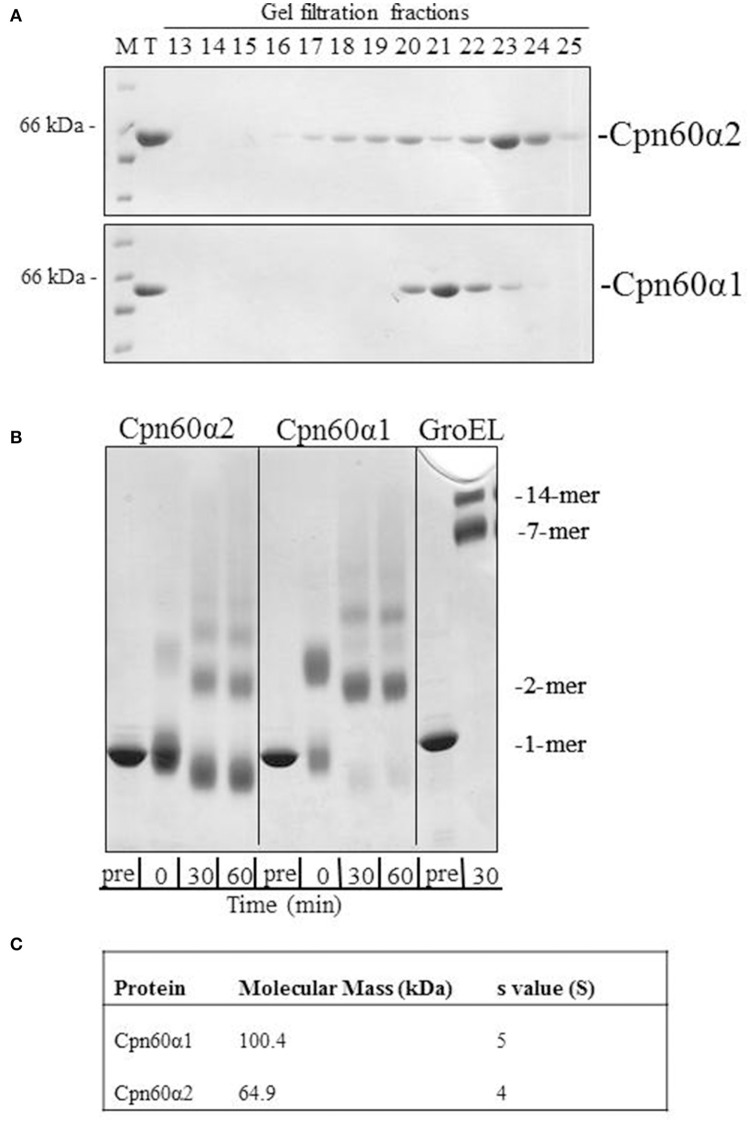
The oligomeric state of Cpn60α1 and Cpn60α2 subtypes. **(A)** Elution profile of α1 and α2 from Superdex 200 gel filtration column. 1 mg protein was injected into a Superdex 200 gel filtration column pre-equilibrated with 50 mM Tris-HCl pH 8, 300 mM NaCl, 5% (v/v) glycerol and run at a rate of 1 ml/min for 120 min. Fractions of 3 ml were collected. Five microliters of each fraction was run on an SDS-PAGE mini-gel. M, molecular weight marker; T, total. **(B)** Cross linking pattern of α1 and α2. Twenty micromolar of purified protein was subjected to cross-linking with 0.1% glutaraldehyde, for the indicated times at RT, in a buffer containing 50 mM Na-HEPES pH 8, 10 mM MgCl_2_ and 100 mM KCl. Samples were analyzed by SDS-PAGE in a 2.4–12% gradient gel and stained with Coomassie Brilliant Blue. **(C)** Analytical ultracentrifugation values for α1 and α2. The data was obtained as described in the Materials and Methods section in buffer: 50 mM Tris-HCl pH 8, 200 mM NaCl, 10 mM MgCl_2_ and 100 mM KCl.

We have cloned and purified both Cpn60α homologs using a strategy that was developed and described for Cpn60β subunits (Vitlin et al., 2011). The final step of the purification process hinted at the physico-chemical differences between these two proteins. As can be seen in Figure 1A and Figure S1A, α1 eluted from the gel filtration column earlier than α2, suggesting that the α1 form is larger than α2. In addition, α1 eluted as a single sharp peak, while the α2 elution profile displayed several peaks. In order to further investigate these differences, we subjected the proteins to crosslinking with glutaraldehyde, to analyze their oligomeric state. The crosslinking pattern of both subunits exhibited several high molecular-weight bands (Figure 1B and Figure S1B). However, one major difference stood out between the α1 and the α2 samples: while the main band in the α2 samples represented the monomeric form, no monomer was observed for α1, but rather a lower mobility species consistent with that of a dimer.

In order to determine the molecular weight of these proteins, we carried out analytical ultracentrifugation. Two variations of this method, sedimentation velocity and sedimentation equilibrium, were used to analyze the α homologs. Using sedimentation velocity, we found that both subunits were characterized by a single peak, with average sedimentation coefficients of ~4 S and 5 S for α2 and α1, respectively. The sedimentation co-efficient of α2 is similar to the previously published coefficient for GroES (70 kDa), 4 S (Seale et al., 1996), indicating that this subunit is mainly monomeric while α1 is larger and most likely a dimer. Analysis of sedimentation equilibrium (Figure 1C) corroborated this observation, with a calculated molecular weight of 100,400 Da for α1 and of 64,900 Da for α2. The expected monomer weight for these proteins is ~57,000 Dalton. Thus, the results of sedimentation equilibrium indicate that α1 is best modeled as a dimer and α2 is primarily monomeric.

### Reconstitution and functional characterization of αβ hetero-oligomers

Since their discovery, chaperonin tetradecamers composed of Cpn60α and Cpn60β subunits have been considered to be the native form of chaperonin oligomers that are active in chloroplasts (Musgrove et al., 1987), although functional β homo-oligomers were described *in vitro* (Dickson et al., 2000; Vitlin et al., 2011; Bai et al., 2015). To our surprise, attempts to reconstitute hetero-oligomers containing α1 were not successful. Reconstitution mixtures containing α1 alone or in combination with any individual β subunit eluted from the gel filtration column as inactive, low molecular weight species (not shown). The fact that no Cpn60β oligomer was formed in the presence of Cpn60α1 was intriguing, since Cpn60β subunits alone generally tend to form oligomers under the same conditions. This suggests that an interaction is taking place between the monomers but it is not productive in furthering formation of a tetradecamer. A similar phenomenon was described for the α subunit of *Chlamydomonas* (Bai et al., 2015), which was incapable of forming mixed oligomers with any individual Cpn60β subunit. Interestingly, in Bai et al. these species composed of one α subunit and one β subunit were still capable of complementing a GroEL deletion strain of *E. coli*.

**Figure 2:**
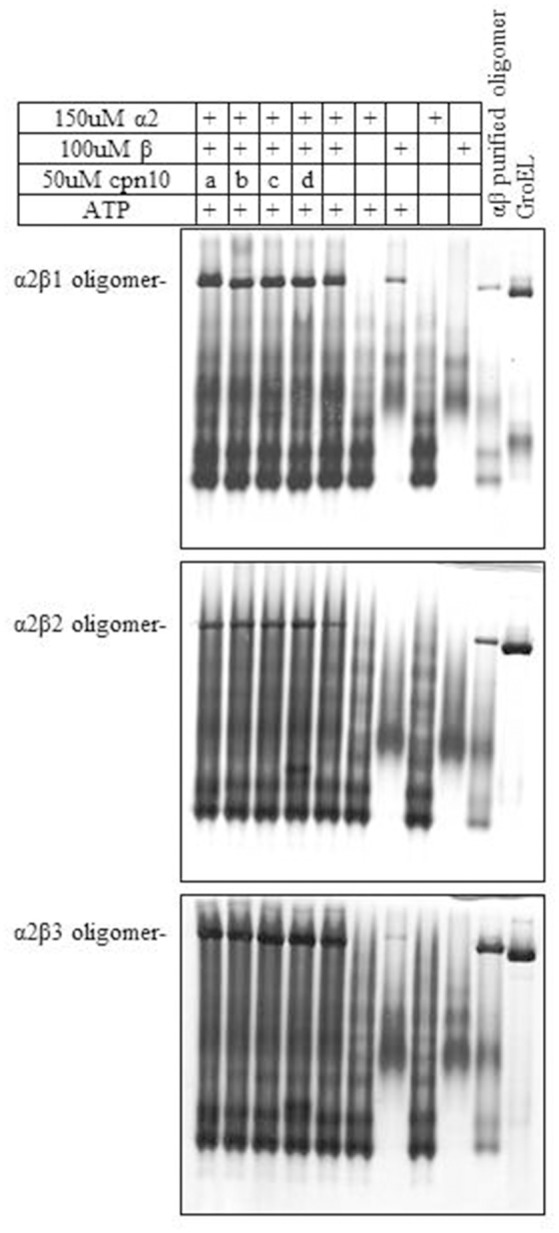
Effect of various Cpn10 homologs on reconstitution of hetero-oligomers. Reconstitution reactions were prepared by incubating 150 μM Cpn60α2 with 100 μM of different Cpn60β subunits and 50 μM of different Cpn10s: a. GroES, b. mt-cpn10, c. Cpn20, d. Cpn10(2), as described in the Materials and Methods section. 1.5 μl of reconstitution product was loaded on a 6% native polyacrylamide gel. Type of oligomeric species formed during the incubation is indicated to the left of the gel.

We next examined the ability of α2 to form mixed oligomers with each of the three β subunits. Initially we followed oligomer formation using native gel electrophoresis. As can be seen in Figure 2 and Figure S2, α2 does not oligomerize on its own, but is able to form mixed oligomers with β1, β2, or β3 homologs. The oligomerization was induced in the presence of Mg^2+^-ATP, however, the presence of different Cpn10s slightly improved the reconstitution efficiency, as was shown previously for Cpn60s from other plant, animal and bacterial sources (Dickson et al., 2000; Bai et al., 2015).

**Figure 3:**
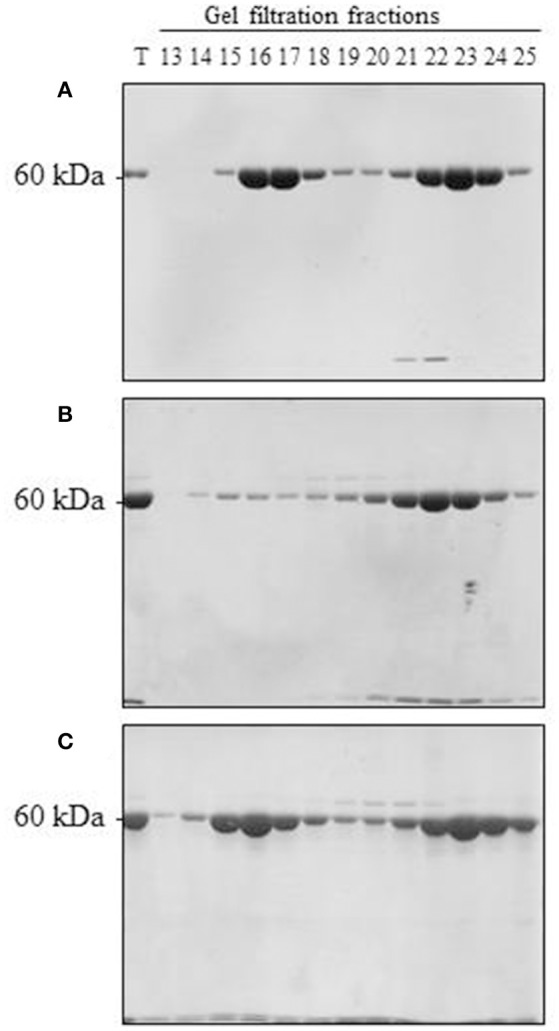
Reconstitution of α2β2 hetero-oligomers and β2 homo-oligomers. **(A)** 150 μM α2, 100 μM β2, and 50 μM mt-cpn10 were reconstituted in a 2 ml reaction mix as indicated in the Materials and Methods section. Samples were then loaded on a Superdex 200 gel filtration column and run at 4°C. Fractions were analyzed by 12 % SDS PAGE and Coomassie Blue staining (7.5 μl was loaded per lane). *T* = 0.25 μl of reconstitution mixture. Oligomer is found in fractions 14–18, monomer is found in fractions 19–25. **(B)** 300 μM β2 and 150 μM mt-cpn10 were reconstituted in a 500 μl reaction and separated by Superdex 200 at 4°C as described in **(A)**. Ten microliters of each fraction was loaded per lane. **(C)** 300 μM β2 and 150 μM mt-cpn10 were reconstituted in a 600 μl reaction and separated by Superdex 200 at room temperature as described in **(B)**.

Upon scaling up the oligomerization process, we considered several additional factors. On the one hand, Cpn60α and Cpn60β subunits were shown to be organized in the oligomer in an ~1:1 ratio (Musgrove et al., 1987; Nishio et al., 1999; Dickson et al., 2000). On the other hand, we wanted to ensure that no self-oligomerization of Cpn60β would occur in the reconstitution experiment (when we prepared the mixed Cpn60α2β oligomers). Initially, we were not able to exclude the possibility that a small amount of Cpn60β homo-oligomer was formed during the oligomerization process, together with the hetero-oligomer. The most significant result of this section was the fact that α2β2 hetero-oligomers were found to be stable when separated using gel filtration at 4°C (Figure 3A and Figure S3A). This is in comparison to β2 homo-oligomers, which dissociate to monomeric form when exposed to the same temperature (Figure 3B and Figure S3B), yet remain stable at room temperature (Figure 3C and Figure S3C), as reported in Vitlin et al. (2011). This enabled us to ensure homogeneity of the α2β2 hetero-oligomer preparation. Since α2β2 was the only hetero-oligomer for which we could guarantee a homogeneous preparation, we focused our efforts on α2β2 oligomers and carried out the reconstitution reactions at an excess of Cpn60α2 and at 4°C.

**Figure 4:**
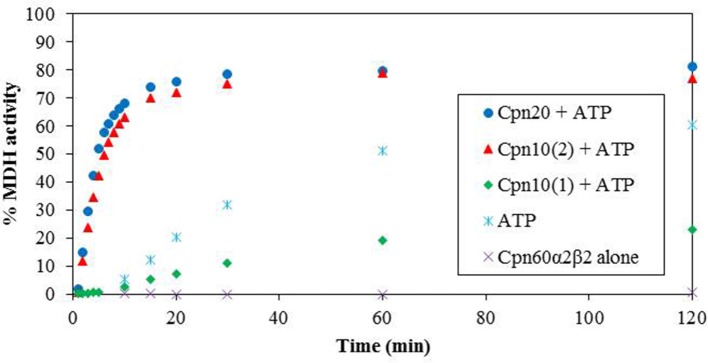
Refolding of denatured MDH by reconstituted α2β2 hetero-oligomers. MDH refolding was carried out by α2β2 hetero-oligomer as described in the Materials and Methods section in 37°C. MDH activity was determined at various time points following the addition of ATP and various Cpn10 homologs. Cpn20: (*filled circle*), Cpn10(2): (*filled triangle*), Cpn10(1): (*filled diamond*), ATP alone: (*asterisk*), α2β2 hetero-oligomer alone: (multi-sign). One hundred percent was taken as the activity of a sample containing a similar amount of native MDH. Values represent the average of two independent experiments.

We next examined the chaperonin activity of the α2β2 hetero-oligomers. As demonstrated in Figure 4 and Table 1, the activity of this hetero-oligomer in the presence of chloroplast co-chaperonins [Cpn10(2) and Cpn20] was similar to that of GroEL and reached the maximal yield of ~80%. It can be seen that α2β2 hetero-oligomer was equally functional with both chloroplast co-chaperonins examined and they both had similar effects on the rate (*t*_1/2_ = 4–5 min) as well.

**Table 1:** Rates and yields of MDH refolding by αβ hetero-oligomers in the presence of chloroplast co-chaperonins^*^.

		**Cpn20**	**Cpn10(2)**
t_½_ (min)	α2β2	4	4.5
	GroEL	4.5	5
Final refolding yields (%)	α2β2	79.8	78.8
	GroEL	74.7	74.7

* *Data extracted from Figure 4 and average of three experiments with GroEL*.

An interesting observation regarding α2β2 hetero-oligomers is the time dependent accumulation of active MDH, observed in the presence of ATP alone without the addition of any co-chaperonin. This is most likely explained by the low stability of the α2β2 oligomer at this concentration, in the presence of destabilizing ATP and the absence of stabilizing co-chaperonin, resulting in dissociation to Cpn60 monomers and release of partly folded MDH, which spontaneously reaches the native folded state as time passes.

We tested the activity of an additional *Arabidopsis* co-chaperonin, Cpn10(1), which was recently characterized (Vitlin Gruber et al., 2014). As can be seen in Figure 4, Cpn10(1) alone is not functional with α2β2 hetero-oligomer. This is consistent with the published results with GroEL and α2β3 hetero-oligomer, where Cpn10(1) was shown to be active only as part of hetero-oligomer with Cpn20 (Vitlin Gruber et al., 2014). Although no protein folding activity is observed, Cpn10(1) has some stabilizing effect on the α2β2 oligomeric structure, indicating that an interaction is taking place between chaperonin and co-chaperonin. Cpn10(1) presence in addition to ATP seems to prevent the hetero-oligomer from dissociating to monomers, thus MDH is not released into the solution and spontaneous folding is not detected as it is in the presence of ATP alone. A similar phenomenon was observed in Bonshtien et al. (2009), where Cpn20 from *Arabidopsis* demonstrated similar binding to β homo-oligomers and αβ hetero-oligomers from pea, yet was unable to facilitate refolding of substrate protein with the β homo-oligomers.

## Discussion

In this study, we have cloned and purified both types of Cpn60α subunits from *A. thaliana* chloroplast. During the characterization of these subunits, we showed that α1 forms mainly dimers in solution, while α2 formed several low molecular weight oligomeric forms in solution. Neither of these alpha species showed any ability to refold urea-denatured MDH. Monomeric and dimeric forms of Cpn60 are found in a number of bacterial species. For example, evidence of a low molecular weight Cpn60 protein complex exists in *Mycobacterium tuberculosis*, which crystallizes as a dimer (Qamra et al., 2004; Shahar et al., 2011). However, in contrast to the *Arabidopsis* α1, the protein from *M. tuberculosis* exhibits some protein folding activity *in vitro*, oligomerizes to higher order forms in the presence of ammonium sulfate, KCl and ATP, and can replace GroEL *in vivo*, suggesting that the functional form *in vivo* is an oligomer (Fan et al., 2012). Similarly, in cyanobacteria, the GroEL1 protein seems to form unstable, yet functional tetradecamers, while the GroEL2 protein remains monomeric. Both of these species exhibit a low level of protein-refolding activity, which does not depend upon GroES and ATP (Reviewed in Nakamoto and Kojima, 2017). In general, the chloroplast α and β chaperonin subtypes are both thought to have evolved from bacterial GroEL1.

In all studies of chloroplast chaperonins thus far, homologs of the α subunits were incapable of self-assembly to tetradecamers. The foundation for chaperonin oligomerization was consistently shown to be one or more of the β subunits. For *Chlamydomonas* chaperonins, this ability was determined to lie in residues of the equatorial domain and part of the intermediate domain (Zhang et al., 2016). Likewise, for type II chaperonins, it was demonstrated that only CCT4 and CCT5 out of the eight subunits, were capable of oligomerizing on their own, or facilitating oligomerization of a hetero-oligomer (Sergeeva et al., 2013).

In contrast to α1, α2 monomers easily formed mixed oligomers with all types of β subunits tested. An important achievement of this work was our ability to ensure that reconstituted α2β2 hetero-oligomers were not contaminated by β2 homo-oligomers. These pure hetero-tetradecamers were equally and maximally active with authentic *Arabidopsis* chloroplast co-chaperonins: Cpn20 and Cpn10(2). Comparison between the activity pattern of α2β2 hetero-oligomer and the β2 homo-oligomer as published in Vitlin et al. (2011), once again assured us that we are dealing with different species with unique patterns of refolding rate and yield. Although Cpn20 could assist both oligomers to reach a maximal yield, Cpn10(2) served as a functional co-chaperonin only with the α2β2 hetero-oligomer and had a very low activity with β2 homo-oligomer.

Only a limited number of *in vitro* studies have been carried out on chloroplast chaperonin proteins. Starting with the early studies of Roy et al. (1988), it was consistently demonstrated that oligomerization is a very dynamic process and oligomer stability is highly concentration dependent. For example, pea chaperonin in chloroplast lysate preparations was shown to dissociate in the presence of ATP when the lysate was diluted 15-fold (Roy et al., 1988). Successful reconstitution was in general shown to require relatively high concentrations of the protein. This is consistent with an estimated chloroplast chaperonin concentration of 175 μM protomer (Lorimer, 1996). For example, urea-dissociated native pea chloroplast chaperonins were successfully reconstituted at a concentration of 60 μM (Lissin, 1995). Reconstitution of αβ hetero-oligomers cloned from pea was carried out using 30 μM of each protein (Dickson et al., 2000). While reconstitution of *Arabidopsis* β1 and β3 homo-oligomer was achieved at over 50 μM protein, β2 was able to form oligomers only at concentrations >200 μM (Vitlin et al., 2011), near the estimated *in vivo* concentration. In addition, Bonshtien et al. (2009) showed that ATPase activity of reconstituted pea chaperonins reached a stable rate only at 60 μM monomer, presumably representing the concentration at which equilibrium favored the oligomeric state.

In addition to protein concentration, temperature is another factor that was shown to significantly affect the stability of organellar chaperonins *in vitro*. Dissociation of pea chaperonin in the presence of ATP or urea was potentiated by lower temperatures (Lissin, 1995; Viitanen et al., 1995). Dissociation at cold temperature was used by Dickson et al., to obtain a uniform population of β monomers as starting material for oligomeric reconstitution (Dickson et al., 2000). Our previous results showed that β2 oligomers are unable to form at 4°C, although significant oligomerization is observed at 25°C under the same conditions (Vitlin et al., 2011). This is also consistent with the behavior of mitochondrial chaperonins, which were demonstrated to be highly unstable in the presence of ATP at 4°C, yet were stable at 37°C under the same conditions (Weiss, 1997).

In conclusion, we demonstrate a method for reconstituting pure hetero-oligomeric chaperonin particles *in vitro* that are free from contaminating homo-oligomers. This method takes advantage of the difference in oligomeric stability between α2β2 and β2 at 4°C. Our results highlight the complex nature of the chloroplast chaperonin system and emphasize how even the simplest physico-chemical conditions must be taken into account when investigating organellar chaperonins *in vitro*.

## Materials and methods

### Nomenclature

In this work, we continue with the nomenclature that was established by Hill and Hemmingsen (2001), and which we previously used for *A. thaliana* chloroplast chaperonin subunits (Weiss et al., 2009; Vitlin et al., 2011; Vitlin Gruber et al., 2013a, 2014). It should be noted that different nomenclature is adopted by other groups.

Cpn60 homologs:

At5g18820 (α1 Cpn60)

At2g28000 (α2 Cpn60)

At5g56500 (β1 Cpn60)

At3g13470 (β2 Cpn60)

At1g55490 (β3 Cpn60)

At1g26230 (β4 Cpn60)

Cpn10 homologs

At3g60210 (Cpn10(1))

At2g44650 (Cpn10(2))

At5g20720 (Cpn20)

### Cloning and purification of chaperonin subunits

Cpn60α1 (At5g18820) and Cpn60α2 (At2g28000) were cloned between the BamHI-NotI sites of a modified version of pET21d+, which codes for an octa-histidine tag followed by the TEV (Tobacco Etch virus) proteolysis site at the amino terminus of the protein (Opatowsky et al., 2003). The first amino acid of the mature protein was chosen based on presequence predictions (Hill and Hemmingsen, 2001): alanine 33 (α1) and alanine 46 (α2). Due to the nature of the cloning, α1 and α2 contained an additional glycine-serine at the N-terminus of the protein. The constructs were expressed in *E. coli* Rosetta (Novagen) and purified based on the Cpn60β purification protocol (Vitlin et al., 2011).

Previously published protocols were used to purify GroES (Bonshtien et al., 2007), Cpn10(1) (Vitlin Gruber et al., 2014), Cpn10(2) (Sharkia et al., 2003), Cpn20 (Bonshtien et al., 2007), mouse mt-cpn10 (Viitanen et al., 1998), Cpn60β1/2/3 (Vitlin et al., 2011) and GroEL (Bonshtien et al., 2007).

### Reconstitution of αβ hetero-oligomers

The reconstitution protocols were based on Vitlin et al. (2011). In short, the experiments were carried out in 50 mM Tris-HCl pH 8, 0.3 M NaCl, 10 mM MgCl_2_, 16 mM KCl, 2 mM dithiothreitol (DTT), 5 mM ATP and different concentrations of Cpn60 and Cpn10 as indicated in the figure legends. The reconstitution mixture was incubated for 5 min at room temperature and then for 1 h at 30°C. For oligomer purification, oligomers and monomers in the reconstitution reaction were separated using a Superdex 200 gel filtration column pre-equilibrated with 50 mM Tris-HCl pH 8, 300 mM NaCl, 5% (v/v) glycerol at 4°C unless stated otherwise. Fractions containing oligomers were pooled, and treated with Ni–NTA-agarose beads in order to remove any traces of his-tagged mt-cpn10 that might have co-purified with the Cpn60. The relevant fractions were concentrated and flash frozen in liquid nitrogen. For oligomerization tests, reconstitution mixtures were run on native 6% polyacrylamide gels.

### Cross-linking

20 μM Cpn60 was cross-linked by 0.1% (v/v) glutaraldehyde (GA—Pierce), at room temperature in 50 mM Na-HEPES (Ph = 7.5), 10 mM MgCl_2_, 100 mM KCl. The cross-linking reaction was stopped by addition of one-third volume of sample buffer: 62.5 mM Tris-HCl pH 6.8, 2% SDS, 5% β-mercaptoethanol, 20% glycerol, 1 M urea. Samples were boiled for 5 min prior to electrophoresis in a large 2.4–12% gradient SDS-PAGE.

### Analytical ultracentrifugation

All experiments were carried out as described in Vitlin Gruber et al. (2013b).

### *In vitro* refolding of urea-denatured MDH

RefoldingA experiments were carried out as described in Vitlin et al. (2011).

## Author contributions

AV, MV, CW, and AA: conceived the ideas and designed experiments; AV, MV, and CW: performed experiments; AV, MV, CW, and AA: analyzed data; AV, CW, and AA: contributed toward writing the manuscript.

### Conflict of interest statement

The authors declare that the research was conducted in the absence of any commercial or financial relationships that could be construed as a potential conflict of interest.
