# Supplementary material for: Reconstitution of Pure Chaperonin Hetero-Oligomer Preparations in Vitro by Temperature Modulation
Source: Front Mol Biosci. 2018 Jan 26;5:5. doi: 10.3389/fmolb.2018.00005 (PMC5790771; doi:10.3389/fmolb.2018.00005)
Supplement: Supplementary file 1 [file Presentation1.ppt]

## Slide 1
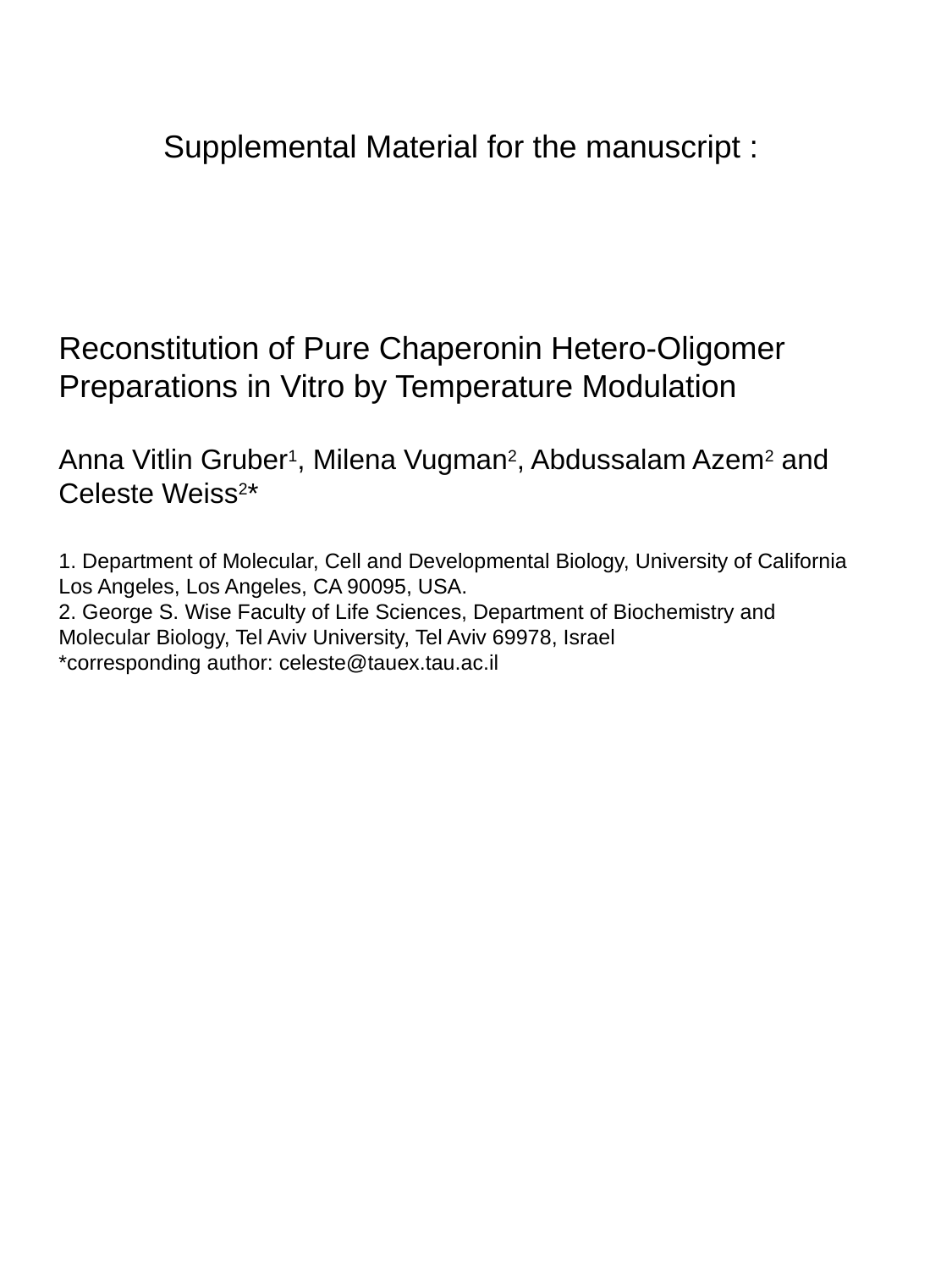

Supplemental Material for the manuscript :
Reconstitution of Pure Chaperonin Hetero-Oligomer Preparations in Vitro by Temperature Modulation
Anna Vitlin Gruber1, Milena Vugman2, Abdussalam Azem2 and Celeste Weiss2*
1. Department of Molecular, Cell and Developmental Biology, University of California Los Angeles, Los Angeles, CA 90095, USA.
2. George S. Wise Faculty of Life Sciences, Department of Biochemistry and Molecular Biology, Tel Aviv University, Tel Aviv 69978, Israel
*corresponding author: celeste@tauex.tau.ac.il

## Slide 2
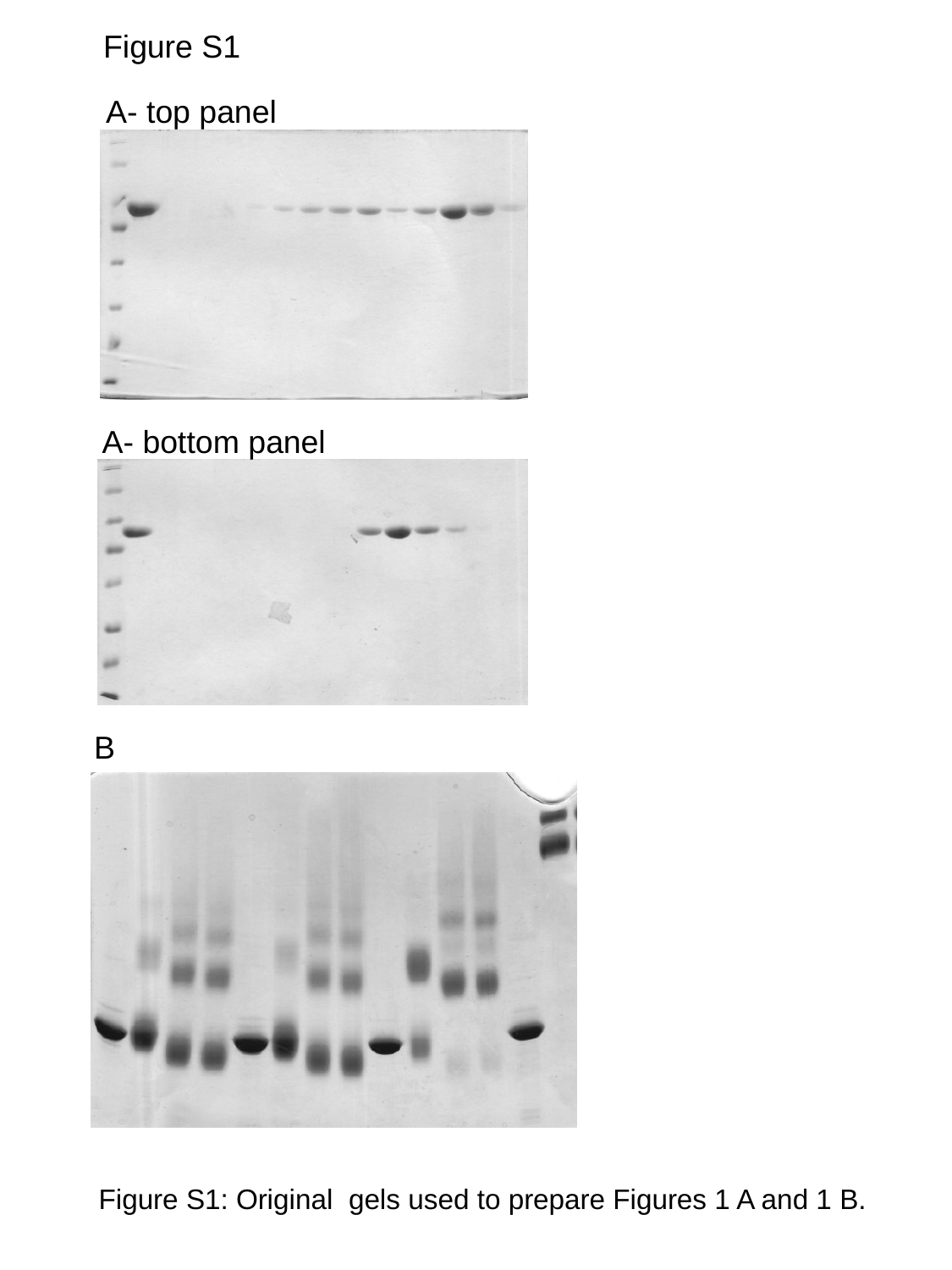

Figure S1
A- top panel
A- bottom panel
B
Figure S1: Original gels used to prepare Figures 1 A and 1 B.

## Slide 3
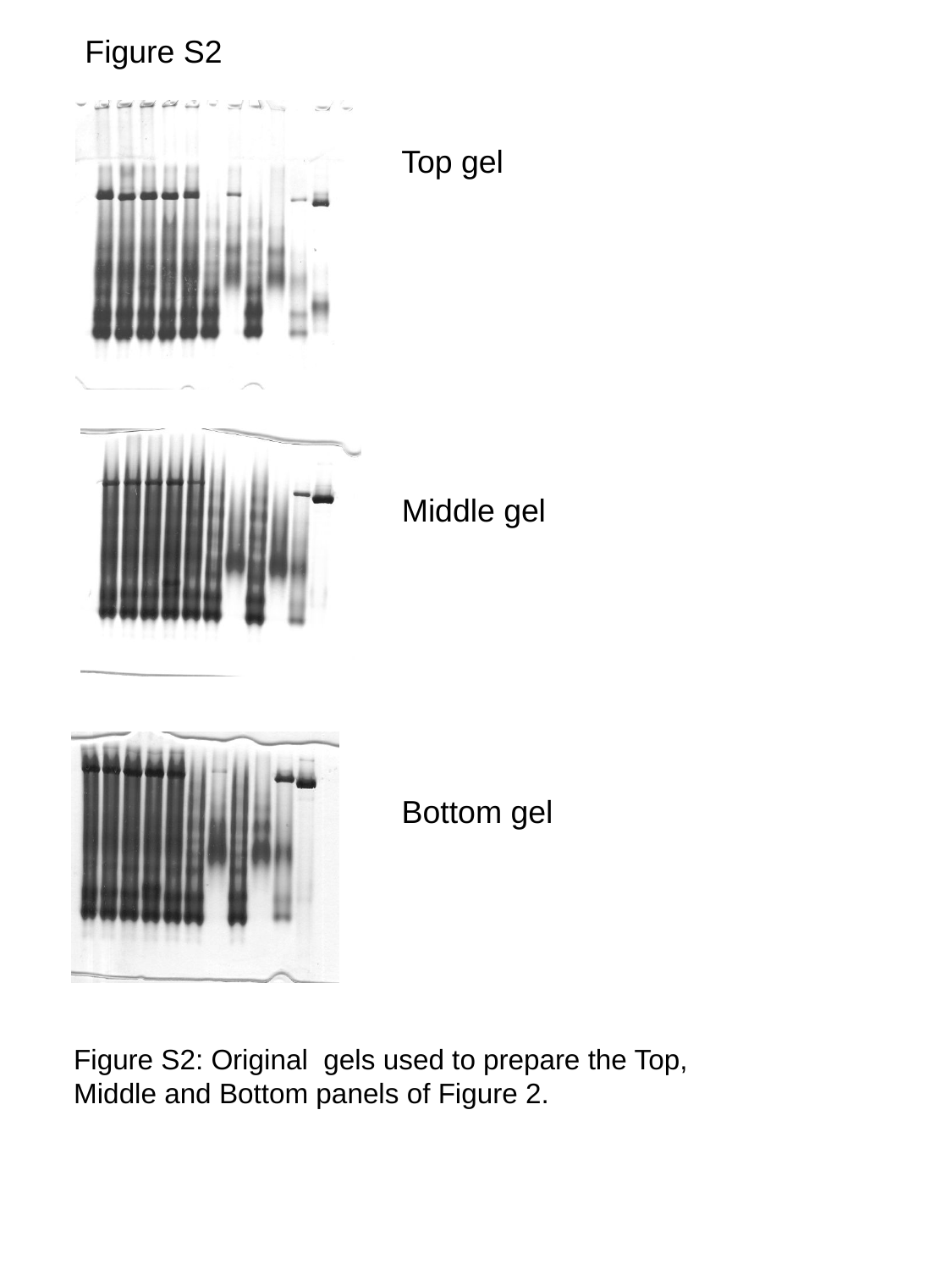

Figure S2
Top gel
Middle gel
Bottom gel
Figure S2: Original gels used to prepare the Top, Middle and Bottom panels of Figure 2.

## Slide 4
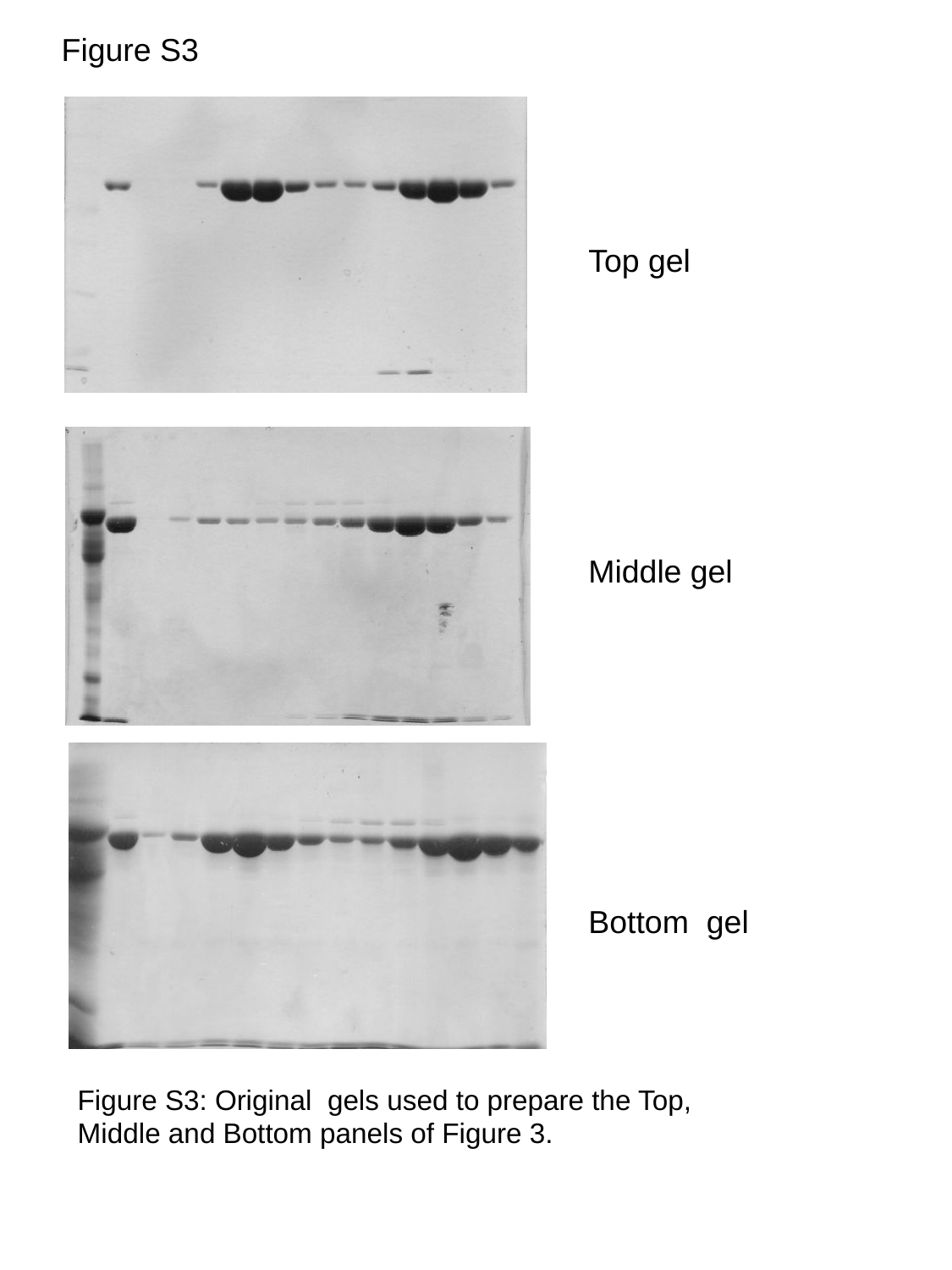

Figure S3
Top gel
Middle gel
Bottom gel
Figure S3: Original gels used to prepare the Top, Middle and Bottom panels of Figure 3.
